# Impact of race/ethnicity and language preferences on pediatric ALL survival outcomes

**DOI:** 10.1002/cam4.5951

**Published:** 2023-04-16

**Authors:** Meghan Davitt, Lisa Gennarini, David Loeb, Melissa Fazzari, H. Dean Hosgood

**Affiliations:** ^1^ Division of Pediatric Hematology, Oncology, Transplant and Cellular Therapy Children's Hospital at Montefiore, Albert Einstein College of Medicine Bronx New York USA; ^2^ Department of Epidemiology and Population Health Albert Einstein College of Medicine Bronx New York USA

**Keywords:** acute lymphoblastic leukemia, racial and ethnic disparities, survival outcomes

## Abstract

**Background:**

Ethnic and racial disparities have recently been observed both in treatment‐related toxicities and rates of long‐lasting cure in acute lymphoblastic leukemia (ALL) and acute lymphoblastic lymphoma (ALLy), the most common pediatric malignancy. Despite significant improvements in overall survival in the recent past, a large number of children die from aggressive disease.

**Methods:**

We performed a retrospective cohort analysis of 274 pediatric ALL/ALLy patients within Montefiore Health System from 2004 to 2021 to determine differences in all‐cause mortality within the Pediatric Hematologic Malignancies Cohort using Cox Proportional Hazard regression modeling, adjusted for age at diagnosis, race/ethnicity, administration of intensive chemotherapy, preferred language, maximum glucose, and hypertension.

**Results:**

Among our 274 patients, 132 were Hispanic, 54 Non‐Hispanic Black, and 25 Non‐Hispanic White, with 25 identified as “Non‐Hispanic Other,” including Asian, Arabic, and Other. Hispanic patients were 78% less likely to die (HR 0.22; 95% CI 0.07, 0.73) when compared with Non‐Hispanic Black individuals. Spanish speakers were 2.91 times more likely to die compared with those who spoke English (HR 2.91; 95% CI 1.08, 7.82). Among those English speakers, the diagnosis of hypertension and Hispanic ethnicity significantly impacted the risk of death, while these factors did not impact survival in Spanish speakers. High‐risk cytogenetics did not impact survival.

**Conclusions:**

Hispanic children with ALL/ALLy have improved survival outcomes compared with Non‐Hispanic Blacks. Additionally, Spanish language preference was strongly associated with poorer survival, a novel finding that should be validated in future studies.

## Graphical abstract

We performed a retrospective cohort analysis of 274 pediatric ALL/ALLy patients within Montefiore Health System from 2004 to 2021 to determine differences in all‐cause mortality within the Pediatric Hematologic Malignancies Cohort using Cox Proportional Hazard regression modeling, adjusted for age at diagnosis, race/ethnicity, administration of intensive chemotherapy, preferred language, maximum glucose, and hypertension. Hispanic patients were 78% less likely to die (HR 0.22; 95% CI 0.07, 0.73) when compared with Non‐Hispanic Black individuals, while Spanish speakers were 2.91 times more likely to die compared with those who spoke English languages (HR 2.91; 95% CI 1.08, 7.82). Hispanic children with ALL/ALLy have improved survival outcomes compared with Non‐Hispanic Blacks, but Spanish language preference was strongly associated with poorer survival, a novel finding that should be validated in future studies.

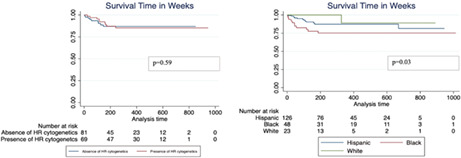

## 1 INTRODUCTION

Ethnic and racial disparities have recently been observed both in treatment‐related toxicities and rates of long‐lasting cure in childhood leukemia and lymphoma.^1^ Acute lymphoblastic leukemia (ALL) is the most common pediatric malignancy, followed by brain tumors and lymphomas.^2^ Outcomes for patients with childhood leukemia and lymphoma have dramatically improved over the past 30 years, with exceptional 5‐year survival rates of >90% in children and adolescents with a newly diagnosed disease, improvements attributed to the use of multiagent systemic chemotherapy, development of broad‐spectrum antibiotics to treat infectious complications, and enhanced availability of supportive blood products.^3^ Despite these improvements, however, a significant number of children continue to die from aggressive disease, and survivors often suffer from lifelong treatment‐related toxicities.^3^ Those who are older at diagnosis or have poor cytogenetic prognostic markers are more likely to suffer from relapsed disease and have reduced survival compared to those with less aggressive disease.^4^, ^5^ More recently, technological advancements and epidemiologic studies have revolutionized the treatment of pediatric patients with hematologic malignancies by enabling providers to tailor therapy for patients based on their individual risk to promote sustained remissions and minimize long‐term chemotherapy‐related morbidity. Patients with ALL and acute lymphoblastic lymphoma (ALLy) are currently risk‐stratified based on age at diagnosis, white blood cell (WBC) count at presentation, cytogenetics, immunophenotype, and evidence of minimal residual disease (MRD) after induction chemotherapy, defined as >0.01% leukemic cells tested using next generation sequencing or flow cytometry.^3^ Those with poor prognostic indicators are stratified into a high‐risk category that requires a more intensive chemotherapy regimen compared with treatment administered to those at a lower risk of relapse.^6^, ^7^ This more intensive chemotherapy regimen leads to a greater potential for durable remission but is accompanied by added risks of both acute and chronic treatment‐related complications.^8^

In addition to the aforementioned high‐risk features, disparities by race/ethnicity and socioeconomic status (SES) have been observed both in treatment‐related toxicities and rates of long‐lasting cure in childhood leukemia and lymphoma, with a paucity of literature analyzing the etiology behind these differences.^1^ Self‐reported race/ethnicity may correlate with SES, while individuals with low SES are at risk for reduced access to high‐quality healthcare, delays in treatment after diagnosis, and difficulty adhering to treatment regimens.^9^, ^10^ Evidence from previous cancer trials suggests that those with lower SES and those of Hispanic ethnicity are more likely to suffer decreased overall survival (OS) and increased rates of relapse.^11^, ^12^, ^13^ Insurance status has also been associated with inferior outcomes, with pediatric cancer patients with public health insurance demonstrating both reduced OS following hematopoietic stem cell transplant and increased risk of death from any cause.^13^, ^14^ Studies that investigate the combined impact of social demographic factors, cytogenetic mutations, and chemotherapy regimens on outcomes in ALL/ALLy patients are currently lacking. Our study sought to evaluate differences in cytogenetic mutations and common chemotherapy‐related toxicities among ALL/ALLy patients of different races/ethnicities to determine whether these predict differential outcomes in all‐cause overall survival.

## 2 METHODS

Patients aged 0–25 years old with ALL/ALLy were identified (*n* = 274) within the Pediatric Hematologic Malignancy Cohort (PHMC), which is a merged retrospective cohort of 180 pediatric patients cared for at the Children's Hospital at Montefiore from 2004–2021 and 413 adult and pediatric aged patients from the Hematological Malignancies Cohort at MMC (HMCMMC),^15^ and includes all lymphoma and leukemia patients diagnosed within the Montefiore Health System (MHS) from 2004–2017. Duplicate patient information was removed based on Medical Record Number (MRN) and date of birth (DOB), and then, demographics, diagnosis date, date of death using National Death Index (NDI), race/ethnicity, preferred language, vital status, biologic sex, DOB, socioeconomic status* (SES), ICD9 and ICD10 diagnosis codes, outpatient prescription data, and data on laboratory variables, including glucose levels, hemoglobin, lipase/amylase, and white blood cell count/neutrophil count were extracted using Montefiore's Electronic Data Warehouse (EDW). Preferred language was determined using data from EDW based on the preference of parents/guardians. We evaluated Body Mass Index percentiles at diagnosis (BMI, percentiles) using WHO guidelines to determine whether those with elevated BMI when diagnosed were more likely to suffer from complications such as hypertension or diabetes. SES was based upon the patient's census block group and is reported as a summary Z‐score relative to the New York State mean using 6 variables with additional details reviewed in Roux et al 2001.^16^ Presence of MRD and high‐risk cytogenetics were determined through retrospective chart review, with confirmation of cytogenetics performed through evaluation of historical logbooks within the Molecular Cytogenetics Laboratory at Albert Einstein College of Medicine. High‐risk cytogenetics were defined as hypodiploid status, *IKZF1* mutation, MLL rearrangement, intrachromosomal amplification of chromosome 21, and Ph+ and Ph‐like genetic mutations.^17^ Data on the presence of MRD were available for 81 patients, while that of high‐risk cytogenetics was available for 158 patients in our analysis.

Patients were classified by features including high‐risk cytogenetics, administration of intensive chemotherapy regimen including doxorubicin at any point in therapy, complications including peripheral neuropathy, hypertension, diabetes, psychosis, cardiomyopathy, febrile neutropenia, and episodes of hyperglycemia and life‐threatening complications including sepsis, pulmonary emboli, deep vein thromboses, and pancreatitis. This was performed in Stata using binary coding for the presence of a complication. Our data are stored as yes/no for all co‐morbidities apart from episodes of hyperglycemia and maximum glucose, which are stored as the total number and number of patients with the reported elevation, respectively. Patients did not have any of these diagnoses prior to diagnosis with ALL/ALLy. ICD9 and 10 codes for each of the diagnoses, along with a review of outpatient prescription records for the drugs related to these complications, were used to identify patients with the listed complications (Table S1). Variables calculating time to complication and time to death from diagnosis were generated. Self‐reported race/ethnicity, extracted from EDW, was used to classify each patient included in PHMC. Language preference was categorized as Spanish or English, with those speaking “Other” omitted from analysis as these accounted for <5% of the patient population.

Population demographics, year of diagnosis, age at diagnosis (years), administration of intensive chemotherapy, time to diagnosis (days), BMI percentiles, SES, preferred language, high‐risk cytogenetics, MRD, and sex were compared by race/ethnicity using Pearson Chi‐square tests, t tests, and ANOVA for normally distributed variables and nonparametric testing for variables not normally distributed evaluated with visual inspection. Kaplan–Meier method and log‐rank test assessed all‐cause mortality and overall survival from diagnosis. All‐cause mortality was defined as the time from the first contact date in EDW to death from any cause and was censored at the date last known alive. Cumulative complication incidence with corresponding 95% confidence intervals by race/ethnicity and high‐risk cytogenetics were evaluated using competing risk regression analysis with death as a competing risk.

Cox proportional hazards regression assessed univariate and multivariable associations between demographic and clinical factors and all‐cause mortality, as well as associations between complications including cardiomyopathy, steroid‐induced diabetes and hypertension, febrile neutropenia, peripheral neuropathy, hyperglycemia, sepsis, deep vein thromboses, pulmonary emboli, and severe necrotizing pancreatitis with race/ethnicity, and high‐risk cytogenetics. Those missing cytogenetic data were removed from model building and evaluation of cumulative incidence. We examined the prevalence of high‐risk cytogenetics within these racial groups using Kaplan–Meier curves and Cox proportional hazard modeling. Log–log plots and Schoenfield residuals were used to assess the proportional hazards assumption for each univariate regression. Hypertension, diabetes, neuropathy, psychosis, cardiomyopathy, febrile neutropenia, sepsis, pancreatitis, inflammatory complications, thrombosis, pulmonary embolus, and deep vein thrombosis were included in the model as time‐varying covariates. Our a priori model included age at diagnosis in years and race/ethnicity. All additional variables that were associated with the outcome in univariate models (*p* < 0.20) were added using stepwise backward elimination to build our final multivariate model, which included age at diagnosis, race/ethnicity, administration of intensive chemotherapy, preferred language, maximum glucose categories, ranging from normal to extreme elevations, and hypertension. Confounders were assessed based on a change in beta >20% and were considered for adjustment in multivariable models. Stratified analyses were performed by race and ethnicity, preferred language, sex, and by absence or presence of high‐risk cytogenetics. All analyses were performed using Stata (Version 17). This study was approved by our Institutional Review Board and was granted a waiver of informed consent due to the nature of the study.

## 3 RESULTS

Among the 274 patients in our cohort, 132 were Hispanic, 54 were Non‐Hispanic Black, and 25 were Non‐Hispanic White, with 25 identified as “Non‐Hispanic Other,” including Asian, Arabic, and Other (Table 1). Median follow‐up time was similar among racial/ethnic groups (*p* = 0.57) (Table 1). Administration of intensive chemotherapy at any point in therapy was increased in Hispanics, compared with Non‐Hispanic Blacks and Non‐Hispanic Whites (69% vs. 61% vs. 40%) (*p* = 0.02). Non‐Hispanic Whites had the highest SES when compared with Hispanics or Non‐Hispanic Blacks (*p* < 0.0001). Among Hispanics, median SES was lower among Spanish speakers compared to those with English language preference (−3.7 vs. −2.8). Year of diagnosis, BMI percentile, sex, presence of high‐risk cytogenetics, white blood cell count at diagnosis (g/dL), MRD at end of induction therapy, and age at diagnosis were similar among racial/ethnic groups (Table 1). A diagnosis of hypertension and the incidence of inflammatory complications, including sepsis, febrile neutropenia, and pancreatitis, were more common in those who were Hispanic (*p* = 0.02; *p* = 0.001, respectively). When adjusting for death as a competing risk, the cumulative incidence of inflammatory complications was associated with race/ethnicity (*p* = 0.03) (Figure S1A). The distribution of patients with serum glucose levels that ranged within normal (<250 g/dL), mildly elevated (250–499 g/dL), moderately elevated (500–749 g/dL), and extremely elevated (>750 g/dL) were similar by race and ethnicity. Similar trends were demonstrated when evaluating the cohort by Hispanic Spanish and Hispanic English speakers, Non‐Hispanic Black English speakers, and Non‐Hispanic White English speakers.

**TABLE 1:** Demographics and clinical characteristics by race and ethnicity from Pediatric Hematologic Malignancies Cohort (PHMC) of 274 patients with Acute Lymphoblastic Leukemia and Lymphoma within Montefiore Health System (MHS)

Demographics	Hispanic (*n* = 132)	Non‐Hispanic Black (*n* = 54)	Non‐Hispanic White (*n* = 25)	*p‐*value
Age at diagnosis, years (median, IQR)	7.0 (3.6, 13.2)	8.1 (4, 15)	10.5 (5.4, 19.3)	0.06
Administration of Intensive Chemo, *n* (%)^a^	91 (69.0)	33 (61.1)	10 (40)	0.02
Follow‐up time, weeks (median, IQR)	218.7 (108.0, 445.0)	260.3 (82.9, 493.9)	124 (45.6, 312.1)	0.57
Diagnosis year (median, IQR)	2013 (2009, 2017)	2011 (2006, 2016)	2014 (2007, 2015)	0.41
Time to diagnosis (mean ± SD)^b^	8.2 ± 21.4	9.0 ± 20.6	8.7 ± 16.7	0.97
Female, *n* (%)	59 (44.7)	23 (42.6)	7.0 (28)	0.66
Body Mass Index (median percentile, IQR)^c^	58 (37, 92)	53 (13, 83)	70 (41, 95)	0.44
Socioeconomic Status^d^, median (IQR)	−3.4 (−6.5, −1.8)	−3.4 (−6.1, −1.2)	0.47 (−1.4, 0.93)	<0.0001
Preferred language spoken, *n* (%)				<0.0001
English	87 (65.9)	52 (96.3)	19 (76)	
Spanish	46 (34.9)	2.0 (3.7)	0 (0)	
White Blood Count at Diagnosis, g/dL (median, IQR)	82.0 (18.0, 121.0)	81.5 (7.2, 112.0)	89.0 (49.0, 89.0)	0.92
Minimal Residual Disease^e^, *n* (%)	11 (28.2)	7 (41.2)	0 (0)	0.44
High‐Risk Cytogenetics, *n* (%)	40 (46.0)	13 (41.9)	5.0 (55.6)	0.77

^a^ Defined by Doxorubicin containing treatment strategies.

^b^ Time to diagnosis defined from first contact to diagnosis date.

^c^ As determined by World Health Organization (WHO) guidelines.

^d^ SES was based upon the patient's census block group and is reported as a summary Z‐score relative to the New York State mean using 6 variables (additional details within manuscript).

^e^ Minimal Residual Disease at End of Induction therapy determined by Flow Cytometry testing at John Hopkins University.

**FIGURE 1:**
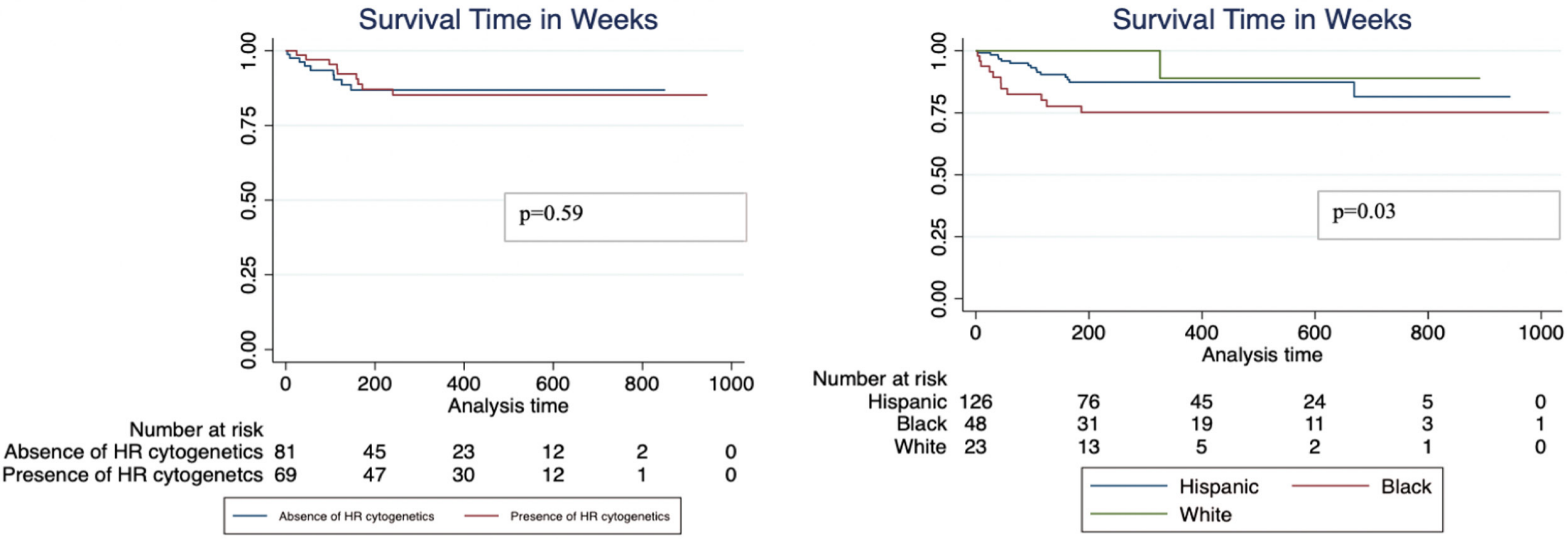
Within 274 patients in Pediatric Hematologic Malignancies Cohort (PHMC) within Montefiore Health System (MHS), survival time in weeks associated with race/ethnicity but not high‐risk cytogenetics

Age at diagnosis, Hispanic ethnicity, insurance status, preferred language, moderate and extreme elevation in blood glucose level (defined as levels >500 and 750 g/dL, respectively), number of episodes of hyperglycemia, MRD, presence of hypertension, diabetes, neuropathy, and pulmonary embolism were all associated with an increased risk of death (Table 2). Non‐Hispanic Blacks had the worst overall survival in our cohort when compared with Non‐Hispanic Whites and Hispanics (*p* = 0.03) (Figure 1). Survival time in weeks was similar when compared among Non‐Hispanic White English speakers, Non‐Hispanic Black English speakers and Hispanic English and Hispanic Spanish speakers, although suggestive that Hispanic English speakers may have had best survival (*p* = 0.26) (Figure S2). Hispanic English speakers had improved overall survival when compared with all others (*p* < 0.0001). Hispanic patients were 78% less likely to die (HR 0.22; 95% CI 0.07, 0.73) when compared with Non‐Hispanic Blacks, while those with Spanish language preference were 2.91 times more likely to die compared with those who spoke English (HR 2.91; 95% CI 1.08, 7.82) (Table 2). Spanish‐speaking Hispanics had a similar risk of death compared with Non‐Hispanic Black English speakers (HR 5.12; 95% CI 0.60, 44.07). Less than half of Hispanics listed Spanish as their preferred language (Table 1).

**TABLE 2:** Within 274 patients in Pediatric Hematologic Malignancies Cohort (PHMC) within Montefiore Health System (MHS), Race/Ethnicity associated with decreased all‐cause mortality, while Spanish Language, Hypertension, Diabetes, and Moderate to Severe Glucose Elevations associated with increased risk of death

	Observations, *n*	Deaths, *n* (%)	HR^h^ (95% CI)	*p‐*value	HR^i^ (95% CI)	*p‐*value
Age at diagnosis (years)	248		1.14 (1.09, 1.19)	<0.0001	1.15 (1.07, 1.25)	<0.0001
Diagnosis year	248		1.02 (0.97, 1.08)	0.47		
Race/Ethnicity						
Non‐Hispanic Black	54	17 (31)	1.00 (ref)		1.00 (ref)	
Hispanic	132	18 (14)	0.49 (0.23, 1.08)	0.08	0.22 (0.07, 0.73)	0.01
Non‐Hispanic White/Other	72	19 (26)	0.94 (0.43, 2.04)	0.87	0.38 (0.11, 1.37)	0.14
Administration of Intensive chemo^a^	248	25 (16)	0.62 (0.34, 1.13)	0.12	0.11 (0.03, 0.45)	0.002
Female Sex			0.62 (0.33, 1.18)	0.15		
Male	152	37 (24)				
Female	112	18 (16)				
Socioeconomic Status^b^	223		0.98 (0.89, 1.09)	0.76		
Insurance Status						
Private/Self Pay	85	14 (17)	1.00 (ref)			
Medicaid	189	41 (22)	2.98 (1.25, 7.08)	0.01		
Preferred language						
Spanish	69	19 (28)	2.24 (1.20, 4.18)	0.01	2.91 (1.08, 7.82)	0.04
English	205	36 (18)	1.00 (ref)		1.00 (ref)	
Body Mass Index (percentile, %)^c^	201		1.01 (1.00, 1.02)	0.23		
Max Glucose Categories						
Normal range (<250)	56	2 (4)	1.00 (ref)		1.00 (ref)	
Mild Elevation (250–499)	47	6 (13)	4.05 (0.82, 20.09)	0.09	8.95 (0.90, 89.40)	0.06
Moderate Elevation (500–749)	22	11 (50)	13.17 (2.73, 63.48)	0.001	28.69 (2.14, 385.24)	0.01
Extreme Elevation (>750)	139	36 (26)	7.51 (1.78, 31.66)	0.006	3.38 (0.41, 28.20)	0.41
Episodes of Hyperglycemia^d^	248		1.04 (1.02, 1.06)	<0.0001		
Minimal Residual Disease^e^	42	5 (25)	1.35 (0.66, 2.75)	0.41		
Presence of High‐Risk Genetics	146	11 (16)	1.15 (0.37, 3.63)	0.81		
Neutropenia^f^	248	32 (20)	1.17 (0.51, 2.67)	0.71		
Sepsis	248	32 (24)	1.89 (0.83, 4.32)	0.13		
Neuropathy	248	11 (26)	2.56 (1.06, 6.19)	0.04		
Hypertension^g^	248	24 (32)	3.17 (1.42, 7.07)	0.005	11.65 (3.04, 44.62)	<0.0001
Diabetes	248	18 (39)	3.49 (1.44, 8.46)	0.006		
Psychosis	248	4 (31)	1.24 (0.17, 9.22)	0.83		
Pancreatitis	248	11 (29)	0.97 (0.33, 2.85)	0.96		
Pulmonary Embolus	248	8 (89)	5.35 (1.82, 15.71)	0.002		
Deep Vein Thrombosis	248	4 (27)	did not converge	>0.99		
Cardiomyopathy	248	1 (33)	did not converge	>0.99		
Inflammatory Complications	248	36 (20)	1.07 (0.46, 2.50)	0.88		
Thrombosis	248	12 (43)	2.22 (0.83, 5.96)	0.11		

*Note*: Model building with a priori inclusion of age at diagnosis ≤25 with all additional variables that were suggested to be associated with the outcome in univariate models^2^ (*p* < 0.20) added using stepwise backward elimination to build our final multivariate model.

^a^ Defined by Doxorubicin containing treatment strategies.

^b^ SES was based upon the patient's census block group and is reported as a summary Z‐score relative to the New York State mean using 6 variables (additional details within manuscript).

^c^ As determined by World Health Organization (WHO) guidelines.

^d^ As determined by Glucose level ≥250 g/dL.

^e^ Minimal Residual Disease at End of Induction therapy determined by Flow Cytometry testing at John Hopkins University.

^f^ Neutropenia defined as Absolute Neutrophil Count ≤500 k/μL.

^g^ Hypertension defined as BP above 95% percentile for age.

^h^ Hazard Ratio (HR) and 95% Confidence Intervals (CI) Determined with Univariate Cox proportional hazard model.

^i^ Hazard Ratio (HR) and 95% Confidence Intervals (CI) Determined with Multivariate Cox proportional hazard model containing 251 observations.

Patients who developed hypertension during therapy were 11.65 times more likely to die when compared to those without hypertension (adjustedHR 11.65; 95%CI: 3.04, 44.62) (Table 2). The effect of hypertension varied based on language preference. Among English speakers, the diagnosis of hypertension alone led to a 7.63‐fold greater risk of death (HR 7.63; 95% CI 2.59, 22.49) (Table S2). This effect was maintained after adjustment for race/ethnicity and administration of intensive chemotherapy. When examining the effect of hypertension across both race/ethnicity and language preference, we observed that hypertension was associated with an increased risk of death in Non‐Hispanic White English speakers (HR 44.39; 95% CI 3.08, 640.18) (Table 3). This impact was not demonstrated in Spanish speakers (Table S2), Hispanic patients (HR 3.03; 95% CI 0.17, 54.22), Non‐Hispanic Black English speakers, Hispanic English speakers, or Hispanic Spanish speakers (Table 3). Cumulative incidence of hypertension was associated with race/ethnicity (*p* = 0.04) (Figure S1B). Sensitivity analyses excluding those of undefined or other race demonstrated similar effects to our main model.

**TABLE 3:** Within 274 patients in Pediatric Hematologic Malignancies Cohort (PHMC) within Montefiore Health System (MHS), Hypertension Associated with Risk of Death in Non‐Hispanic English Speakers

	Non‐Hispanic Black English speaking (*n* = 26)		Non‐Hispanic White English speaking (*n* = 30)		Hispanic English speaking (*n* = 53)		Hispanic Spanish speaking (*n* = 22)	
	HR^a^ (95% CI)	*p‐*value	HR^a^ (95% CI)	*p‐*value	HR^a^ (95% CI)^b^	*p‐*value	HR^a^ (95% CI)^b^	*p‐*value
Age at diagnosis (years)	1.12 (0.95, 1.33)	0.18	1.09 (0.92, 1.28)	0.31	1.24 (0.98, 1.57)	0.08	1.44 (0.99, 2.11)	0.05
Administration of Intensive chemoª	0.71 (0.06, 7.94)	0.78	0.20 (0.03, 1.52)	0.12	0.23 (0.00, 15.38)	0.49	4.91 (0.08, 305.57)	0.45
Hypertension	3.61 (0.33, 39.75)	0.30	44.39 (3.08, 640.18)	0.005	5.27 (0.08, 339.41)	0.43	0.78 (0.07, 9.07)	0.84

^a^ Hazard Ratio (HR) and 95% Confidence Intervals (CI) Determined with Cox proportional hazard model.

^b^ Defined by Doxorubicin containing treatment strategies.

Patients with moderate elevation in blood glucose level (defined as levels between 500 and 749 g/dL) had a 28.69‐fold greater risk of death (HR 28.69, 95% CI 2.14, 385.24) (Table 2). The cumulative incidence of diabetes was similar by race/ethnicity (Figure S1C). Exploratory analyses performed evaluating Hispanics versus Non‐Hispanics and males versus females demonstrated similar effects as our main effects model but did suggest that moderate elevation in glucose levels had a larger impact among Non‐Hispanics (HR 17.89; 95% CI 0.71, 452.25).

We did not find any association between all‐cause mortality and high‐risk cytogenetics in our cohort. Cumulative incidence of inflammatory complications, hypertension, and diabetes was similar between high‐risk cytogenetics groups (Figure S1A–C). We evaluated risk outcomes including hypertension, diabetes, and inflammatory complications and found that high‐risk cytogenetics was not associated with an increased risk of these complications. Those with hypertension were more likely to die in the absence or presence of high‐risk cytogenetics (*p* = 0.02, 0.004, respectively).

## 4 DISCUSSION

In this study, we reviewed how race/ethnicity, SES, and treatment complications impact the survival of pediatric patients diagnosed with ALL/ALLy treated at an inner city academic medical center. We were surprised to find that Spanish language preference significantly impacts survival. As a group, Hispanic patients had improved survival compared with Non‐Hispanic Blacks, and among Hispanic patients, there was a significant improvement in survival among English speakers compared to those with Spanish language preference. Among Hispanics, those speaking English were more at risk of death if hypertensive, while those speaking Spanish did not demonstrate survival differences based on those same factors, indicating that Spanish speakers may be at risk of death due to different factors than their English‐speaking counterparts.

Spanish speakers may be at a unique disadvantage when being treated for ALL/ALLy, possibly due to postdiagnosis disparities, including communication barriers with medical providers. English‐speaking Hispanics may have less of a communication barrier, improving their ability to understand various requirements in the treatment of ALL/ALLy, including medication recommendations, return to care precautions, and treatment schedules for oral chemotherapy, all of which could greatly impact survival. Studies analyzing outcomes in pediatric patients with CNS malignancies demonstrate that overall survival for Hispanic patients is decreased compared with Non‐Hispanic patients, even after adjusting for SES, implying that disparities in survival may be due to lack of access to high‐quality care.^18^ This study suggested that there are postdiagnosis disparities that may account for survival differences in the Hispanic population but did not include language preference as an isolated variable, instead incorporating non‐English language preference as a measure contributing to SES.^18^ Our study evaluated a unique and diverse cohort mostly made up of minority patients, all with low SES cared for within MHS, a tertiary care referral center that cares for patients regardless of ability to pay, yet we still demonstrated differences in outcomes in ALL/ALLy, with Hispanic patients demonstrating improved survival compared with Non‐Hispanic Blacks but with Spanish speakers faring worse. This finding supports the concept that differences in survival may result from postdiagnosis differences, such as lack of understanding of treatment regimens and communication barriers, representing a unique opportunity to improve outcomes for patients with Spanish language preference.

Our results demonstrate a novel finding that Hispanic patients within this ethnically diverse population have a reduced risk of death when compared to their Non‐Hispanic Black counterparts. Hispanic pediatric patients are also more likely to receive intensive chemotherapy with doxorubicin at any point during therapy, despite the finding that multiple poor prognostic factors, including age at diagnosis, white blood cell count at presentation, MRD at end of induction chemotherapy, and presence of high‐risk cytogenetic mutations happen with similar frequency between racial/ethnic groups. This suggests that the administration of intensive chemotherapy including doxorubicin reduces mortality, as those receiving more of this chemotherapeutic have improved survival.

The diagnosis of hypertension, one made commonly during treatment for acute lymphoblastic leukemia and lymphoma,^19^ is associated with an increased risk of death and is more common in those of Hispanic ethnicity. When evaluating the effect of hypertension on the survival of Hispanics, English speakers with hypertension had an increased risk of death that was not seen in Spanish speakers. Similarly, Non‐Hispanics had a 10‐fold increased risk of death if hypertensive, while Hispanics had no increased risk of death if hypertensive. Moderate elevation in glucose levels may also lead to an increased risk of death, with previous studies suggesting that episodes of hyperglycemia may portend an increased risk of hypertension during pediatric ALL/ALLy treatment.^19^ Elevated glucose levels were not associated with an increased risk of hypertension in our cohort but were associated with increased mortality risk along with the development of hypertension, suggesting another area for further study in a larger cohort. With tight control of blood pressures and glucose levels throughout therapy, we may be able to reduce the risk of death during ALL/ALLy therapy, thereby improving outcomes and the risk of long‐term toxicities.

This study examined the role of demographic and clinical prognostic indicators in overall survival and in the incidence of chemotherapy complications in pediatric ALL/ALLy patients within a diverse population cohort. While the sample size and importantly the total number of deaths in our study, only 56 total, was limited, a total cohort of 274 patients does represent a large pediatric cohort within an ethnically diverse population base. However, the chances of overfitting, particularly with a larger multivariable model and within our smaller stratified models, is high, which likely explains the wide confidence intervals demonstrated in our analysis. Our study was only able to evaluate all‐cause mortality due to limitations when reporting the cause of death within NDI, representing another potential limitation of our study. The NDI has been shown to be valid when the cause of death is available^20^; however, up to 73% of deaths in pediatric cancer patients were due to their primary malignancy, with the remainder from competing causes including sepsis, heart disease, and suicide,^21^ making all‐cause mortality an excellent proxy to evaluate survival in pediatric cancer patients. Our study is also limited in that the patient population studied contained a limited number of Non‐Hispanic White patients, with the majority of patients self‐reporting as either Hispanic or Non‐Hispanic Black, limiting the generalizability of findings to Non‐Hispanic White patients. Our patient population, however, represents a unique opportunity to study and determine what places more ethnically diverse patient populations at risk for poor outcomes from ALL/ALLy.

We did not find any associations between high‐risk cytogenetics and survival based on race and ethnicity but did find the suggestion that Spanish speakers with high‐risk cytogenetics have an increased risk of death compared with English speakers. This suggests that communication barriers may impact survival, possibly due to difficulties following guidance from medical providers during treatment, such as fever guidelines or return to clinic precautions, placing patients at risk for infectious complications from treatment and management of ALL/ALLy. Missing cytogenetic data represent a potential limitation of the study, as this aspect of the study may have been underpowered, with limited information available regarding the cytogenetic mutations in our cohort, with model sizes decreased due to less data available on presence or absence of cytogenetic mutations. Because high‐risk cytogenetics was not found to be associated with all‐cause mortality in univariate modeling, we did not include it in our model building since it was not included in our a priori model. We did evaluate the risk of other outcomes including hypertension, diabetes, and inflammatory complications and found that high‐risk cytogenetics was not associated with an increased risk of these complications. Cytogenetic analysis has vastly changed over the past 15 years, with more mutations now identified as portending a higher risk of relapse than initially thought, which may have impacted the ability to study this feature in our cohort. Recent studies, however, have examined the role of cytogenetic aberrations in an effort to understand drivers of relapse and poor outcomes in ALL.^8^ Higher rates of relapse may actually be seen more in patients with MRD at end of initial induction chemotherapy rather than in those with negative MRD, indicating that certain cytogenetic mutations, such as *IKZF1* deletions, alone may not be indicative of poorer outcomes.^8^ We identified a portion of our patients that had evidence of MRD following induction therapy but were unable to determine this for all patients in the cohort, representing a limitation of our study. The debate regarding these cytogenetic markers demonstrates the need for increased research into the treatment strategies and responses to chemotherapy that patients with such mutations have.^8^

A particular strength of our study is the diverse population base within MHS, a safety net hospital system that serves a large historically marginalized minority population within the Bronx, NY, and provides care for all individuals, regardless of insurance status, representing a medical home in which patients can be assured of continued care regardless of financial or social concerns. Although a limitation of this study is that preferred language and self‐reported race/ethnicity were extracted from EDW, which may contain inaccuracies such as incorrect labeling based on provider evaluation, previous studies utilizing EMR‐based data extraction methods have demonstrated accurate results, reaffirming the utility of data extraction methods in retrospective analyses.^22^, ^23^ Use of translators was not able to be extracted from EDW, another limitation of this study. Inadequate interpreter services may lead to inferior quality of care,^24^ and this, along with our finding that Spanish language preference is associated with increased mortality, highlights the need to improve interpreter services in pediatric cancer care. The use of preferred language within the study also allows us to evaluate the Hispanic subset of patients to determine differences in outcomes of ALL based on limited English proficiency while highlighting the need to continue studying this important factor influencing ALL outcomes. We were also unable to evaluate the role of hematopoietic stem cell transplant and post‐therapy BMI percentiles on outcomes in this population due to limitations within the electronic medical record, both of which would be interesting factors to evaluate in future studies.

Hispanics are underrepresented in clinical trials in ALL/ALLy, with those speaking Spanish possibly at a larger disadvantage than those speaking English because some trials lack Spanish informed consent documents. Thus, Spanish‐speaking patients may have been excluded from studies evaluating these regimens, even though census data reports that Hispanics make up the second largest ethnic group after Non‐Hispanic Whites.^25^, ^26^ In some pediatric leukemia clinical trials, disparities have been demonstrated in degrees of enrollment as well, with Hispanic and Non‐Hispanic Black patients enrolled to a lesser degree than Non‐Hispanic White patients.^25^, ^27^ Winestone et al demonstrated that early mortality was actually decreased among patients enrolled on a clinical trial, compared with those unenrolled.^27^ If fewer Spanish‐speaking patients were studied on clinical trials, the mortality risk for Spanish speakers may be secondary to being unenrolled on clinical trials within MHS or from the limited understanding gained from previous clinical trials that may have included fewer Spanish speakers. Other studies have demonstrated a reduced OS for Hispanic patients and found through genome‐wide studies a high proportion of Native American ancestry without evaluation of specific ancestries known to contribute to Hispanic ethnicity, including Central American.^28^ The genetic makeup of our cohort was not evaluated, but previous cohort studies within the Bronx suggest a high prevalence of Puerto Rican and Dominican ancestry,^29^ making genetic differences a less likely contributor to improved survival. Our study emphasizes the need to further study this unique population to understand what places them at greater risk of death and chemotherapy complications from treatment of pediatric ALL/ALLy.

In summary, this study has demonstrated that pediatric Hispanic patients with ALL/ALLy have improved survival compared with Non‐Hispanic Blacks and suggests that language preference has a larger impact on survival than previously recognized. Further analysis is needed to confirm these findings. Our analysis suggests the need for further resources to reduce postdiagnosis disparities regarding access to high‐quality care, with a specific focus on reducing language barriers and improving medical literacy within a historically underserved community.

## AUTHOR CONTRIBUTIONS

**Meghan Davitt:** Conceptualization (lead); data curation (lead); formal analysis (lead); funding acquisition (lead); methodology (lead); software (lead); visualization (lead); writing – original draft (lead); writing – review and editing (lead). **Lisa Gennarini:** Conceptualization (supporting); data curation (supporting); investigation (supporting); resources (supporting); supervision (supporting); writing – review and editing (supporting). **David Loeb:** Conceptualization (supporting); resources (supporting); writing – review and editing (supporting). **Melissa Fazzari:** Data curation (supporting); formal analysis (supporting); validation (supporting); writing – review and editing (supporting). **H. Dean Hosgood:** Conceptualization (supporting); data curation (lead); formal analysis (supporting); funding acquisition (supporting); methodology (supporting); resources (lead); supervision (lead); visualization (supporting); writing – review and editing (supporting).

## FUNDING INFORMATION

The research described was supported in part by NIH/National Center for Advancing Translational Science (NCATS) Einstein‐Montefiore CTSA Grant Number UL1TR001073 and the Montefiore Einstein Cancer Center Support Grant Number 2P30CA013330 and Children's Hospital at Montefiore Fellow Research Award.

## CONFLICT OF INTEREST STATEMENT

None to declare.

## ETHICS APPROVAL

Approved by our Institutional Review Board and granted waiver of informed consent.

## Supporting information


Data S1:

## Data Availability

Deidentified data will be made available to other investigators as requested via email without unreasonable restrictions.

## Endnote

* SES was based upon the patient's census block group and is reported as a summary Z‐score relative to the New York State mean using 6 variables (additional details in following reference: [16]. Roux AVD, Merkin SS, Arnett D, Chambless L, Massing M, Nieto FJ, et al. Neighborhood of Residence and Incidence of Coronary Heart Disease. New England Journal of Medicine. 2001;345(2):99–106.)
