# Supplementary material for: Impact of race/ethnicity and language preferences on pediatric ALL survival outcomes
Source: Cancer Med. 2023 Apr 16;12(11):12827–36. doi: 10.1002/cam4.5951 (PMC10278473; doi:10.1002/cam4.5951)
Supplement: Supplementary file 1 — Data S1: [file CAM4-12-12827-s001.docx]

**Supplementary Material Table of Contents**

**Supplemental Table 1 (Page 2):** Determination of complication incidence utilizing Electronic Medical Record (EMR) ICD coding.

**Supplemental Table 2 (Page 3):** Within 274 patients in Pediatric Hematologic Malignancies Cohort (PHMC) within Montefiore Health System (MHS), Hispanic ethnicity, minimal residual disease (MRD), hypertension, diabetes, moderate elevation in glucose, pulmonary embolus associated with all-cause mortality in English speakers only

**Supplemental Figure 1 (Page 4):** Within 274 patients in Pediatric Hematologic Malignancies Cohort (PHMC) within Montefiore Health System (MHS), Cumulative Incidence of inflammatory complications in weeks (2a), Cumulative incidence of hypertension in weeks (2b), and Cumulative Incidence of Diabetes in weeks (2c) adjusting for death as competing risk by high-risk cytogenetics and Race/Ethnicity

**Supplemental Figure 2 (Page 5):** Within 274 patients in Pediatric Hematologic Malignancies Cohort (PHMC) within Montefiore Health System (MHS), Survival time in weeks similar between Hispanic Spanish, Hispanic English, Non-Hispanic Black and Non-Hispanic White English speakers.

**Supplemental Table 1. Determination of complication incidence utilizing Electronic Medical Record (EMR) ICD coding.**

| Diagnoses | ICD 10/9 diagnosis code |
| --- | --- |
| Acute Lymphoblastic Leukemia/Lymphoma | C91.00, 91.01/204.00, 204.01 |
| Pancreatitis | K85/577.0 |
| Deep Vein Thrombosis | I82, I82.40/435.40 |
| Pulmonary Embolus | I26/415.1 |
| Steroid induced Hypertension | I15, I10/401.1 |
| Diabetes | E09,E13/249.0-249.9 |
| Sepsis | R65.2/785.52, 995.92 |
| Febrile neutropenia | D70.9/288.00 |
| Peripheral neuropathy | G62.0/357.6 |
| Cardiomyopathy | I42.7/425.9 |
| Steroid induced psychosis | F19.959/292.89 |

**Supplemental Table 2.** **Within 274 patients in Pediatric Hematologic Malignancies Cohort (PHMC) within Montefiore Health System (MHS), Hispanic ethnicity, minimal residual disease (MRD), hypertension, diabetes, pulmonary embolus associated with all-cause mortality in English speakers only**

|  | **English speakers (n=149)** |  | **Spanish speakers (n=66)** |  |
| --- | --- | --- | --- | --- |
|  | **HR^1^ (95% CI)** | ***p-*value** | **HR^1^ (95% CI)** | ***p-*value** |
| **Age at diagnosis (years)** | 1.14 (1.07, 1.20) | <0.0001 | 1.22 (1.12, 1.33) | <0.0001 |
| **Race/Ethnicity** |  |  |  |  |
| **Non-Hispanic Black** | 1.00 (ref) |  | 1.00 (ref) |  |
| **Hispanic** | 0.18 (0.05, 0.71) | 0.01 | 0.60 (0.16, 2.24) | 0.45 |
| **Non-Hispanic White/Other** | 0.56 (0.18, 1.78) | 0.33 | did not converge |  |
| **Administration of Intensive chemoª** | 0.89 (0.32, 2.50) | 0.82 | 0.18 (0.05, 0.69) | 0.01 |
| **Body Mass Index (percentile, %)^b^** | 1.02 (1.00, 1.03) | 0.05 | 1.00 (0.98, 1.01) | 0.60 |
| **Max Glucose Categories** |  |  |  |  |
| **Normal range (<250)** | 1 (ref) |  | 1 (ref) |  |
| **Mild Elevation (250-499)** | 4.56 (0.47, 43.90) | 0.19 | did not converge |  |
| **Moderate Elevation (500-749)** | 9.97 (0.90, 110.11) | 0.06 | did not converge |  |
| **Extreme Elevation (>750)** | 5.26 (0.67, 41.53) | 0.12 | did not converge |  |
| **Episodes of Hyperglycemia^c^** | 1.10 (1.05, 1.16) | <0.0001 | 0.98 (0.81, 1.19) | 0.85 |
| **Presence of High Risk Genetics** | 1.15 (0.37, 3.63) | 0.81 | 6.57 (0.73, 58.95) | 0.09 |
| **Minimal Residual Disease^d^** | 7.21 (1.19, 43.53) | 0.03 | did not converge |  |
| **Neutropenia^e^** | 2.19 (0.70, 6.90) | 0.18 | 0.33 (0.09, 1.21) | 0.09 |
| **Sepsis** | 3.17 (1.01, 9.96) | 0.05 | 0.85 (0.23, 3.16) | 0.80 |
| **Neuropathy** | 5.72 (2.03, 16.14) | 0.001 | 0.42 (0.05, 3.36) | 0.41 |
| **Hypertension^f^** | 7.63 (2.59, 22.49) | <0.0001 | 0.48 (0.10, 2.33) | 0.36 |
| **Diabetes** | 6.42 (2.32, 17.80) | <0.0001 | did not converge |  |
| **Psychosis** | 5.44 (0.70, 42.32) | 0.11 | did not converge |  |
| **Pancreatitis** | 1.67 (0.47, 5.93) | 0.43 | 0.25 (0.03, 2.00) | 0.19 |
| **Pulmonary Embolus** | 6.13 (1.73, 21.76) | 0.005 | 3.92 (0.48, 31.96) | 0.20 |
| **Deep Vein Thrombosis** | did not converge |  | did not converge |  |
| **Cardiomyopathy** | did not converge |  | did not converge |  |
| **Inflammatory Complications** | 1.69 (0.54, 5.32) | 0.37 | 0.34 (0.09, 1.27) | 0.11 |
| **Thrombosis** | 2.57 (0.72, 9,12) | 0.14 | 1.29 (0.27, 6.24) | 0.75 |
| ^1^Hazard Ratio (HR) and 95% Confidence Intervals (CI) Determined with Cox proportional hazard model | | | | |
| ªDefined by Doxorubicin containing treatment strategies | |  |  |  |
| ^b^as determined by World Health Organization (WHO) guidelines | | |  |  |
| ^c^ as determined by Glucose level ≥ 250 g/dL | |  |  |  |
| ^d^Minimal Residual Disease at End of Induction therapy determined by Flow Cytometry | | | |  |
| ^e^Neutropenia defined as Absolute Neutrophil Count ≤ 500 k/μL | |  |  |  |
| ^f^Hypertension defined as BP above 95% percentile for age | |  |  |  |

**Supplemental Figure 1. Within 274 patients in Pediatric Hematologic Malignancies Cohort (PHMC) within Montefiore Health System (MHS), Cumulative Incidence of inflammatory complications in weeks (2a), Cumulative incidence of hypertension in weeks (2b), and Cumulative Incidence of Diabetes in weeks (2c) adjusting for death as competing risk by high-risk cytogenetics and Race/Ethnicity**


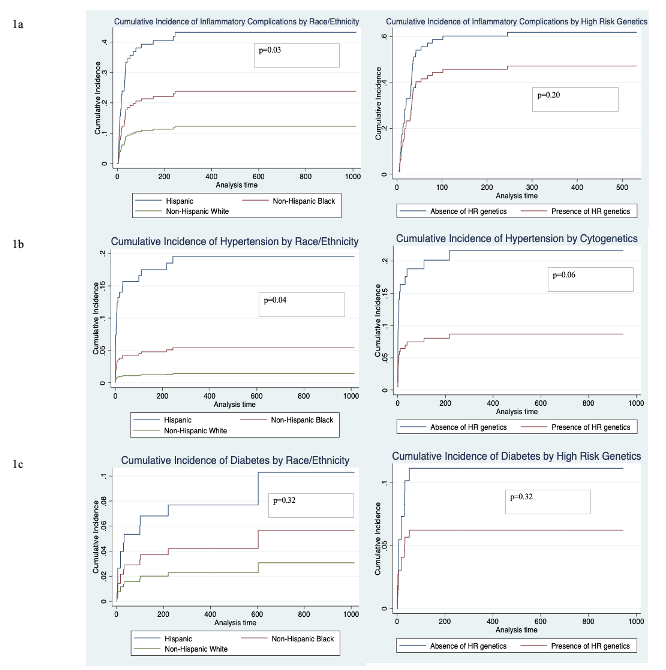


**Supplemental Figure 2. Within 274 patients in Pediatric Hematologic Malignancies Cohort (PHMC) within Montefiore Health System (MHS), Survival time in weeks similar between Hispanic Spanish, Hispanic English, Non-Hispanic Black and Non-Hispanic White English speakers.**

**
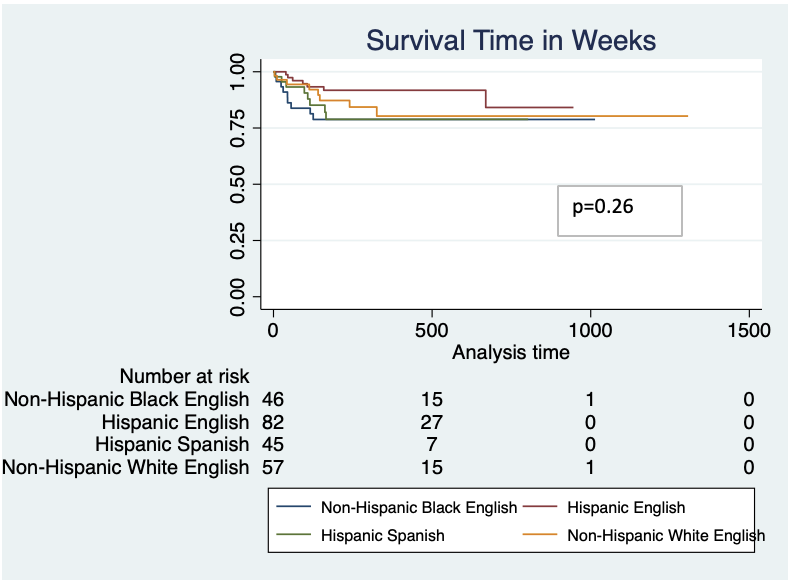
**
